# Associations among workplace environment, self-regulation, and domain-specific physical activities among white-collar workers: a multilevel longitudinal study

**DOI:** 10.1186/s12966-018-0681-5

**Published:** 2018-05-31

**Authors:** Kazuhiro Watanabe, Norito Kawakami, Yasumasa Otsuka, Shigeru Inoue

**Affiliations:** 1 Department of Mental Health, Graduate School of Medicine, The University of Tokyo, 7-3-1 Hongo, Bunkyo-ku, Tokyo, 113-0033 Japan; 2 Faculty of Human Sciences, University of Tsukuba, 3-29-1, Otsuka, Bunkyo-ku, Tokyo, 112-0012 Japan; 3 Department of Preventive Medicine and Public Health, Tokyo Medical University, 6-1-1 Shinjuku, Shinjuku-ku, Tokyo, 160-8402 Japan

**Keywords:** Physical activity, Workplace environment, Multilevel study

## Abstract

**Background:**

Psychological and environmental determinants have been discussed for promoting physical activity among workers. However, few studies have investigated effects of both workplace environment and psychological determinants on physical activity. It is also unknown which domains of physical activities are promoted by these determinants. This study aimed to investigate main and interaction effects of workplace environment and individual self-regulation for physical activity on domain-specific physical activities among white-collar workers.

**Methods:**

A multi-site longitudinal study was conducted at baseline and about 5-month follow-up. A total of 49 worksites and employees within the worksites were recruited. Inclusion criteria for the worksites (a) were located in the Kanto area, Japan and (b) employed two or more employees. Employee inclusion criteria were (a) employed by the worksites, (b) aged 18 years or older, and (c) white-collar workers. For outcomes, three domain-specific physical activities (occupational, transport-related, and leisure-time) at baseline and follow-up were measured. For independent variables, self-regulation for physical activity, workplace environments (parking/bike, signs/bulletin boards/advertisements, stairs/elevators, physical activity/fitness facilities, work rules, written policies, and health promotion programs), and covariates at baseline were measured. Hierarchical Linear Modeling was conducted to investigate multilevel associations.

**Results:**

Of the recruited worksites, 23 worksites and 562 employees, and 22 worksites and 459 employees completed the baseline and the follow-up surveys. As results of Hierarchical Linear Modeling, stairs/elevator (*γ*=3.80 [SE=1.80], *p*<0.05), physical activity/fitness facilities (*γ*=4.98 [SE=1.09], *p*<0.01), and written policies (*γ*=2.10 [SE=1.02], *p*<0.05) were significantly and positively associated with occupational physical activity. Self-regulation for physical activity was associated significantly with leisure-time physical activity (*γ*=0.09 [SE=0.04], *p*<0.05) but insignificantly with occupational and transport-related physical activity (*γ*=0.11 [SE=0.16] and *γ*=−0.00 [SE=0.06]). Significant interaction effects of workplace environments (physical activity/fitness facilities, work rules, and written policies) and self-regulation were observed on transport-related and leisure-time physical activity.

**Conclusions:**

Workplace environments such as physical activity/fitness facilities, written policies, work rules, and signs for stair use at stairs and elevators; self-regulation for physical activity; and their interactions may be effective to promote three domain-specific physical activities. This study has practical implications for designing multi-component interventions that include both environmental and psychological approaches to increase effect sizes to promote overall physical activity.

## Background

The need for promotion of physical activity had been strongly recognized among workers since rapid changes to the modern labor market have resulted in a large increase of workers engaged in sedentary behavior and low-activity occupations [1]. Physical activity is one of the most important health behaviors in public health [2], and promoting this behavior is one of the most evident workplace interventions for primary prevention of common mental disorders [3]. Moreover, significant associations between physical activity and work-related outcomes have repeatedly been reported, such as well-being and presenteeism [4], absenteeism, job stress, employee turnover [5], and work ability [6]. These findings indicate that promoting workers’ physical activity is indispensable for occupational health promotion and a sustainable workforce.

To promote physical activity among workers, multilevel factors across different levels have been suggested as determinants and targets for intervention [7, 8]. Of the psychological determinants, self-efficacy and self-regulation for physical activity are recognized as important factors in theoretical models [9–11]. Especially, self-regulation (e.g., planning, scheduling, and self-organisational behaviors) has recently been indicated to be strongest mediator between physical activity interventions and behavioral changes in physical activity in a non-clinical adult population [12]. Environmental determinants have also been discussed for promoting physical activity [13]. Workplace environment can largely influence activities among workers [14]. A workplace environment for promoting physical activity typically includes organising assessments/counseling/educations, informational support (e.g., posters/flyers/bulletin boards/maps), organisational policy, internal physical environment (e.g., physical activity equipment/stairs/lockers/showers/office connectivity), co-workers’ social support, and external environment (e.g., walkability/parking/active commuting/physical activity facilities outside the workplace) [15–17]. These environments might be effective for promoting not only physical activities during work but also activities out of work (e.g., transport-related and leisure-time). Several observational studies suggested that the effects of workplace environment spilled over to leisure-time physical activity and entire lifestyle [18–20]. Thus, workplace environment is also an important determinant for physical activity among workers.

However, few studies have investigated effects of both workplace environment and psychological determinants on physical activity among workers using a multilevel study design, and no study has dealt with self-regulation for physical activity. It is also unknown which domains of physical activities are promoted by workplace environment and psychological determinants, since most studies measured only overall physical activity or one specific domain of activities. It is important to stratify the domains of physical activity because each activity could arise in different contexts, could be caused by different determinants [21], and could influence different outcomes [22]. Previous observational studies among three large worksites in Canada indicated that self-efficacy for physical activity mediated the relationship between perceived workplace environment and physical activity in the workday [23, 24]. However, in these studies, physical activity was measured only during the workday. Furthermore, they analysed the associations using simple regression as opposed to multilevel analyses and did not address self-regulation. Another previous study was a multilevel longitudinal study among 16 worksites and 129 employees in Japan [25], which showed that employers providing fitness facilities increased the positive association between self-efficacy for physical activity and overall physical activity. However, they did not measure specific domains of activities and did not address self-regulation either. For a clearer understanding of how workplace environment, psychosocial determinants, and physical activity interact, further studies are needed.

In this study, we aimed to investigate the associations among workplace environment, self-regulation for physical activity, and three domain-specific physical activities—occupational, transport-related, and leisure-time—in white-collar workers using a multi-worksite longitudinal design. White-collar workers are mainly engaged in sedentary work and are a primary target among the working population [19]. Our hypotheses were as follows: (*H1*) several aspects of supportive workplace environment will be positively associated with each domain of physical activity among white-collar workers; *(H2)* self-regulation for physical activity will be positively associated with higher scores in each domain of physical activity among white-collar workers; *(H3)* two-level interaction effects of workplace environment and self-regulation for physical activity will be positively associated with higher scores in each domain of physical activity among white-collar workers. This means that the positive association between self-regulation of white-collar workers and higher scores in each domain of physical activity will be stronger under well-facilitated workplace environments.

## Methods

### Study design and setting

This was a multi-site longitudinal study. Multilevel nested data were collected twice—at baseline (Oct–Dec 2015) and at follow-up (Feb–Apr 2016) —from worksites and workers employed by the worksites. We approached worksites in the Kanto area, the economic center of Japan including metropolitan Tokyo, through some of the health insurance associations and chambers of commerce in the area, using snowball sampling methods. Each worksite was explained the study in detail and asked to join the study. After the worksite representatives agreed to partake in the study, nested employees were recruited. Informed consent was obtained from all representatives and workers via a consent form and the self-report questionnaire. The consent form and questionnaire informed participants that we assured protection of personal information and that the data would be anonymous and only used for academic purposes. The study protocol received ethical approval by the research ethics committee of the Graduate School of Medicine and Faculty of Medicine, The University of Tokyo, Japan (No. 10919). Our study has been reported according to the Strengthening the Reporting of Observational studies in Epidemiology (STROBE) guidelines [26].

### Participants

At baseline, a total of 49 representatives of worksites were provided with an explanation of the study and asked to participate by the corresponding author. Worksite inclusion criteria (a) were located in the Kanto area, the economic center of Japan including metropolitan Tokyo, and (b) employed two or more employees. There were no exclusion criteria for worksites. Within the worksites which agreed to partake in the study, nested employees were recruited. Employee inclusion criteria were (a) employed by the worksites, (b) aged 18 years or older, and (c) white-collar workers (managerial, professional, technical clerical, and other job types that required deskwork or sitting work). There were no specific exclusion criteria.

### Measures

We measured worksite- and employee-level variables at the baseline survey and measured physical activity both at the baseline and the follow-up survey. Worksite-level variables were measured by a validated measurement, which consisted of observations by two independent raters from the research team and a self-report questionnaire for worksite representatives (usually human resource personnel). Employee-level variables were measured by a self-report questionnaire distributed to workers.

#### Workplace environment

Workplace environment to promote physical activity was measured by the Japanese version of the Environmental Assessment Tool (EAT) [25, 27]. In this study, workplace environment was operationally defined as EAT scores. Higher scores on the EAT indicate a more supportive environment for physical activity promotion and more invested environment by employers [28]. The concurrent validity with the rate of health abnormality and test–retest reliability of the EAT were confirmed in previous studies in both the US and Japan [25, 28]. In this study, subordinate components of the EAT that were used in the previous study [25] and suggested positive association with physical activity from the literature review [16, 17, 29–32] were used: parking/bike (4 points; e.g., bike rack spaces), signs/bulletin boards/advertisements (4 points; e.g., signs with physical activity messages), stairs/elevator (4 points; e.g., signs encouraging stair use at building entrance or at elevators), physical activity/fitness facilities (14 points; e.g., onsite fitness facilities), work rules (6 points; e.g., own lockers employees can have), written policies (6 points; e.g., written policies supporting employees’ physical activity), and health promotion programs (20 points; e.g., education classes for physical activity). Scores of health promotion programs for physical activity, diet/nutrition, and weight management were combined into one variable to avoid complexity in models. These scores were calculated by using the EAT scoring system based on a representative-reported questionnaire (section 1) and an observation form completed by researchers (section 2). Scores of the observation (section 2) were rated by two of seven trained members from our research team for each worksite and averaged. The research team members were graduate students in psychology, nursing, public health, and health science. Inter-rater reliability of the EAT scores ranged from to .46 to 1.00, considered sufficient values.

#### Self-regulation for physical activity

Self-regulation for physical activity was measured by the Japanese version of the 12-item Physical Activity Self-Regulation scale (PASR-12) [33, 34]. The PASR-12 asked the workers how often they had utilized cognitive and behavioral methods for physical activity in the past four weeks (e.g., “I mentally kept track of my physical activity”). The 12-item measure consists of six factors: self-monitoring, goal-setting, eliciting social support, reinforcements, time management, and relapse prevention. All items are rated on a 5-point Likert scale (1 = *Never*, 5 = *Very Often*). Internal consistency, convergent validity, and structural validity of the Japanese version of the PASR-12 were confirmed in a previous study [33]. In the present study, the total scale score was used in analyses to avoid complexity. Cronbach’s alpha for the scale at the baseline survey was .93, considered an excellent value.

#### Physical activity

Three domain-specific physical activities were measured by the Japanese version of the Global Physical Activity Questionnaire (GPAQ v2) [35]. This scale is widely used and has demonstrated reliability and convergent validity in nine countries, including Japan [36]. The test-retest reliability in each domain was moderate to strong both in the validation study (Spearman’ rho 0.67 to 0.81) [36] and in this study (Intra-Class Correlation 0.66 to 0.84). The GPAQ can assess occupational, transport-related, and leisure-time physical activity with regard to intensity of the activity (moderate-to-vigorous) and time spent doing the activity (minutes, hours, days) based on a typical week. In the present study, the amounts of three domain-specific physical activities per week (METs-hours/week) were calculated according to the GPAQ analysis guide [37]. Participants who responded with inconsistent answers or implausible values were excluded from all analyses in the study (e.g., more than seven days in any column of days spent doing the activity per week).

#### Covariates

Worksite-level confounders included worksite size and worksite location (urban, suburban, and local). Worksite size was operationalized as an ordinal variable and classified into four categories (10–49, 50–99, 100–299, and ≥300 employees). Worksite location was measured by a single ordinal item (urban, suburban, and rural) for worksite representatives, “Where is the worksite located?”

Self-efficacy for physical activity as another psychological determinant, gender, age, employment status (regular, part-time, dispatched, contract, and others), occupational status (managerial, technical and professional, clerical, and others), and working hours per week (1–34, 35–40, 41–50, 51–60, 61–65, 66–70, and ≥71 hours) were measured as employee-level confounders. Self-efficacy was measured using a scale developed by Marcus et al. [38] and Oka [39]. Because the original scale was developed to assess self-efficacy for exercise, we revised the word “exercise” to “physical activity.” The scale consists of four items (e.g., “I have the confidence to perform physical activity even if I am a little tired”). All items are rated on a 5-point Likert-type scale (1 = *Not at All*, 5 = *Almost*). Internal consistency and unidimensional structural validity were confirmed in a previous study [39]. In the present study, the total score of self-efficacy for physical activity was tripled to match its score range to that of self-regulation for physical activity (12–60) to compare the effect sizes on physical activity in multilevel models. Cronbach’s alpha for the scale at baseline survey was .87, considered an excellent value.

Additionally, job strain as determinants for physical activity was assessed. Job strain was measured by the Japanese version of the Job Content Questionnaire 22-item version (JCQ) [40, 41]. The JCQ includes five items for job demands (e.g., “My job requires working very fast”) and nine items for job control (e.g., “I have a lot of say about what happens on my job”). All items are rated on a 4-point Likert-type scale (1 = *Strongly disagree*, 4 = *Strongly agree*). The scale has been widely used to assess job strain, and its reliability and validity were confirmed by a previous study [40]. Cronbach’s alphas for job demands and job control at the baseline survey were .65 and .75, respectively. According to the user’s guide for the JCQ [41], we calculated continuous scores of job strain by the ratio of job demands to job control.

### Analyses

Descriptive statistics, intra-class correlation coefficients for employee-level variables, and multilevel correlation coefficients for two-level variables were calculated. For the main analysis, Hierarchical Linear Modeling (HLM) was conducted to investigate multilevel relationships among workplace environment, self-regulation for physical activity, and physical activity in each domain. Three domain-specific physical activities at follow-up were entered into the models as the dependent variables. Workplace environment, self-regulation for physical activity at baseline, and interaction effects of workplace environment and self-regulation for physical activity were entered as independent variables. Worksite size, worksite location, self-efficacy for physical activity, gender, age, employment status, job type, working hours, and job strain at baseline were controlled as the covariates. In addition, physical activities at baseline were also controlled for to analyze these associations longitudinally. The categorical covariates were transformed into dummy variables: worksite size (10–49 [reference group]), worksite location (urban [reference group]), gender (men [reference group]), employment status (regular [reference group]), occupational status (not managerial [reference group]), and working hours per week (≤ 40 hours [reference group]). Of the continuous variables, worksite-level variables (workplace environment) were grand-mean centered, and employee-level variables (self-regulation and self-efficacy for physical activity, age, job strain, and each domain of physical activity at baseline) were group-mean centered for the multilevel analysis to make the correlations between two-level variables equal to zero [42].

We estimated an unconditional model (model 1), a crude random slope model (model 2), a crude interaction model (model 3), an employee-level adjusted model (model 4), and a worksite-level adjusted model (model 5) in HLM and referred to the Akaike Information Criteria (AIC) as a fit index. The equation for the adjusted model (model 5) was explained as follows.

Level 1 (employee-level)

$$ Y{\left( each\ domain\ of\ physical\ activtiy\  at\  follow- up\right)}_{ij}={\beta}_{0j}+{\beta}_{1j}\ast {\left( self- regulation\right)}_{ij}+{\beta}_{2j}\ast {\left( self- efficacy\right)}_{ij}+{\beta}_{3j}\ast {(gender)}_{ij}+{\beta}_{4j}\ast {(age)}_{ij}+{\beta}_{5j}\ast {\left( Not\ regular\ employment\right)}_{ij}+{\beta}_{6j}\ast {\left( Managerial\  job\right)}_{ij}+{\beta}_{7j}\ast {\left(\geqq 41\  working\ hours\  per\  week\right)}_{ij}+{\beta}_{8j}\ast {\left( job\  strain\right)}_{ij}+{\beta}_{9j}\ast {\left(\  each\ domain\ of\ physical\ activity\  at\  baseline\right)}_{ij}+{e}_{ij} $$

Level 2 (worksite-level)

$$ {\beta}_{0j}={\gamma}_{00}+{\gamma}_{01}\ast {(parking)}_j+{\gamma}_{02}\ast {(signs)}_j+{\gamma}_{03}\ast {(stairs)}_j+{\gamma}_{04}\ast {\left( fitness\ facilities\right)}_j+{\gamma}_{05}\ast {\left( work\ rules\right)}_j+{\gamma}_{06}\ast {(policies)}_j+{\gamma}_{07}\ast {\left( health\ promotion\ programs\right)}_j+{\gamma}_{08}\ast {\left( work site\ scale\ 50-99\right)}_j+{\gamma}_{09}\ast {\left( work site\ scale\ 100-299\right)}_j+{\gamma}_{010}\ast {\left( work site\ scale\ge 300\right)}_j+{\gamma}_{011}\ast {\left( local\ or\ suburban\ area\right)}_j+{\mu}_{0j} $$

$$ {\beta}_{1j}={\gamma}_{10}+{\gamma}_{11}\ast {(parking)}_j+{\gamma}_{12}\ast {(signs)}_j+{\gamma}_{13}\ast {(stairs)}_j+{\gamma}_{14}\ast {\left( fitness\ facilities\right)}_j+{\gamma}_{15}\ast {\left( work\ rules\right)}_j+{\gamma}_{16}\ast {(policies)}_j+{\gamma}_{17}\ast {\left( health\ promotion\ programs\right)}_j+{\mu}_{1j} $$

$$ {\beta}_{qj}={\gamma}_{q0}\ \left(q=2\dots 9\right)\kern2.5em \left(\begin{array}{c}{\mu}_{oj}\\ {}{\mu}_{1j}\end{array}\right)\sim N\ \left[\left(\begin{array}{c}0\\ {}0\end{array}\right)\left(\begin{array}{cc}{\tau}_{00}& {\tau}_{01}\\ {}{\tau}_{10}& {\tau}_{11}\end{array}\right)\right] $$

Because the Full Information Maximum Likelihood (FIML) method was used for parameter estimation to avoid a selection bias due to dropout and to impute missing variables, the worksites and employees that partially had missing values or that dropped out during follow-up were included in the analysed model. We describe the number of missing samples on all variables used in the analyses in descriptive statistics. Mplus version 7.4 [43] (Muthén & Muthén, 1998–2015) was used for each analysis.

We treated three domain-specific physical activities as the continuous values (METs-hours/week). Although distribution of these scores was right-skewed and might not be suitable assuming normal distribution, estimating cross-level interaction effects between individual and workplace was so complex in the multi-level model; improper solutions were observed when we handled the outcomes as ordinal values. In addition, ordinal outcomes were not applied in our model because we applied FIML.

## Results

**Fig. 1:**
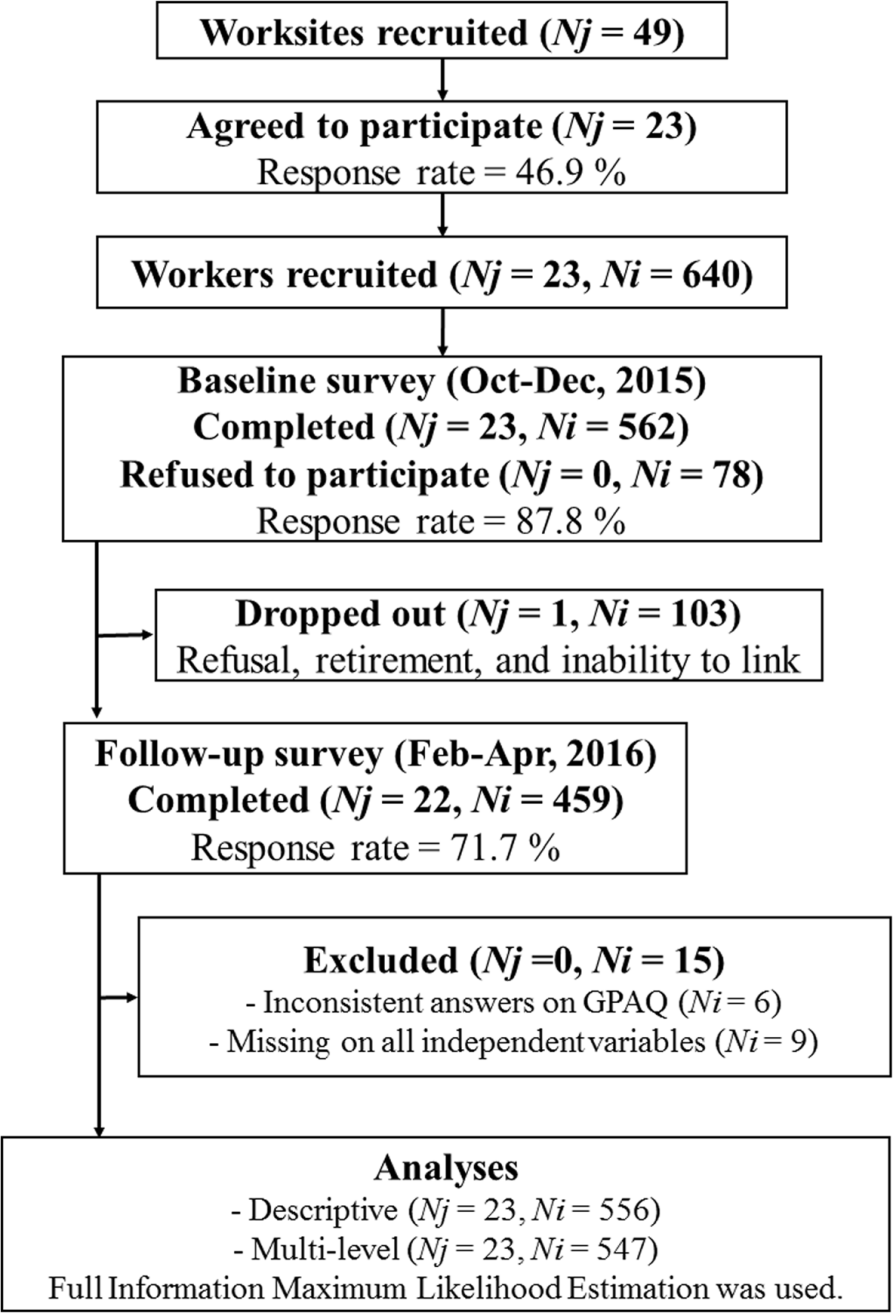
A participation flow chart of the worksites and the workers. Note. *Nj* = the number of worksites; *Ni* = the number of workers

Figure 1 shows a participation flow chart of the worksites and the employees in the study. Of 49 representatives of the worksites, 23 representatives agreed and signed a consent form (response rate = 46.9%). Of the worksites and employees, 23 worksite representatives and 562 employees (265 men, 293 women, and 4 unknown; mean age = 43.5, *SD* = 11.1) completed the baseline survey (response rate = 87.8 %). The average number of workers who completed the baseline survey from each worksite was 24.4 (*SD* = 22.3). At the follow-up, 22 worksite representatives and 459 employees (227 men, 229 women, and 3 unknown; mean age = 44.7, *SD* = 11.1) completed the survey (response rate = 71.7 %). One worksite and 103 workers refused or dropped out during follow-up because of being too busy or unable to link.

Table 1 shows characteristics of the worksites and employees at baseline (23 worksites and 556 employees). Of 562 employees, 6 were excluded due to inconsistent answers on the questionnaire to measure physical activity [35, 37]. Among the worksites, 12 (52.2%) employed 10–49 workers, classified as small-size worksites. The other 11 worksites employed 50–99 (6), 100–299 (3), and more than 300 workers (2). Most of the worksites (60.9%) were located in an urban area (e.g., 23 special wards in Tokyo) in the Kanto region of Japan. Types of industries were service (nine worksites), manufacturing (four), medical and welfare (three), information and communication (three), education (two), wholesale and retail (one), and transportation (one). Of the employees, most (69.8%) were employed full-time. About half (52.4%) of the employees were engaged in clerical jobs, 112 (20.4%) mainly had managerial jobs, and 73 (13.1%) were engaged in technical and professional jobs. A total of 340 (62.0%) employees worked over 40 hours per week, considered long-working workers [44]. Between employees who completed the follow-up survey and who dropped out, dropped-out employees were significantly younger than the completers (*p* = 0.001). There was no other significant difference between two groups for demographic, exposure, and outcome variables.

**Table 1:** Characteristics of the worksites and employees at baseline (Nj = 23, Ni = 556)

Worksite-level variables (*Nj = 23*)			*N* (%)	Min–Max	Mean (SD)	Missing (%)	
Worksite size						0 (0.0)	
10–49			12 (52.2)	–	–		
50–99			6 (26.1)	–	–		
100–299			3 (13.0)	–	–		
≥300			2 (8.7)	–	–		
Worksite location				–	–	0 (0.0)	
Urban			14 (60.9)	–	–		
Local/suburban			9 (39.1)	–	–		
Workplace environment						0 (0.0)	
Parking/Bike			–	0.00–3.00	1.28 (1.0)		
Signs/Bulletin boards/Advertisements			–	0.00–4.00	1.15 (1.2)		
Stairs/Elevator			–	0.00–2.00	0.74 (0.6)		
Physical activity/Fitness facilities			–	0.00–7.00	0.74 (1.7)		
Work rules				3.00–6.00	5.04 (0.8)		
Written policies			–	0.00–6.00	0.26 (1.3)		
Health promotion programs			–	0.00–6.00	1.43 (1.9)		
Employee-level variables	Total (*Ni* = 556)		Follow-up completer (*Ni* = 454)		Drop-out (*Ni* = 102)		*p* for difference
	N (%) *Mean (SD) [Min–Max]*	Missing (%)	N (%) *Mean (SD) [Min–Max]*	Missing (%)	N (%) *Mean (SD) [Min–Max]*	Missing (%)	
Demographic variables							
Gender		4 (0.7)		2 (0.4)		2 (2.0)	0.091
Male	263 (47.6)		223 (49.3)		40 (40.0)		
Female	289 (52.4)		229 (50.7)		60 (60.0)		
Age	*M=43.56 (11.1) [19–84]*	12 (2.2)	*M=44.27 (11.1) [19–84]*	6 (1.3)	*M=40.21 (10.2) [21–65]*	6 (5.9)	0.001
Employment Status		3 (0.5)		0 (0.0)		3 (2.9)	0.980
Regular	386 (69.8)		317 (69.8)		69 (69.7)		
Non-regular (Part-time, contract, dispatched)	167 (30.2)		137 (30.2)		30 (30.3)		
Occupational status		6 (1.1)		3 (0.7)		3 (2.9)	0.926
Clerical	288 (52.4)		236 (52.3)		52 (52.5)		
Managerial	112 (20.4)		94 (20.8)		18 (18.2)		
Technical and professional	73 (13.1)		59 (13.1)		14 (14.1)		
Others	77 (14.0)		62 (13.7)		15 (15.2)		
Working hours per week		8 (1.4)		3 (0.7)		5 (4.9)	0.850
≤40 hours	208 (38.0)		172 (38.1)		36 (37.1)		
≥41 hours	340 (62.0)		279 (61.9)		61 (62.9)		
Job stressors							
Job strain (job demands by job control)	*M=0.48 (0.1) [0.20–1.00]*	19 (3.4)	*M=0.48 (0.1) [0.20–1.00]*	15 (3.3)	*M=0.48 (0.1) [0.20–1.00]*	4 (3.9)	0.849
Psychological determinants							
Self-regulation for physical activity	*M=24.66 (10.1) [12–57]*	9 (1.6)	*M=24.56 (10.1) [12–57]*	4 (0.9)	*M=25.09 (9.9)* [12–50, 53, 54]	5 (4.9)	0.640
Self-efficacy for physical activity	*M=11.45 (3.6)* [4–20]	4 (0.7)	*M=11.46 (3.6)* [4-20]	3 (0.7)	*M=11.44 (3.8)* [4–20]	1 (1.0)	0.954
Physical activity (METs-hours/week)							
Occupational	*M=3.21 (18.7) [0–304]*	5 (0.9)	*M=3.42 (19.8) [0–304]*	4 (0.9)	*M=2.31 (12.7) [0–124]*	1 (1.0)	0.590
Transport-related	*M=10.98 (16.5) [0–144]*	6 (1.1)	*M=10.72 (16.1) [0–144]*	5 (1.1)	*M=12.12 (18.4) [0–140]*	1 (1.0)	0.442
Leisure-time	*M=7.89 (14.6) [0–124]*	5 (0.9)	*M=8.17 (14.3) [0–124]*	4 (0.9)	*M=6.64 (15.8) [0–124]*	1 (1.0)	0.344

*Nj* the number of worksites, *Ni* the number of employees.

After excluding nine participants due to missing data on all independent variables, multilevel correlation coefficients among workplace environment, self-regulation and self-efficacy for physical activity, and each domain of physical activity among 547 employees are shown in Table 2. Intra-class correlation coefficients for domain-specific physical activities ranged from 0.04 to 0.15, indicating 4–15% of the variances were explained by these worksite-level variables. For the employee-level psychological determinants, leisure-time physical activity was most strongly associated with self-regulation and self-efficacy for physical activity (*γ* = 0.40, *p* < 0.01 and *γ* = 0.34, *p* < 0.01), and transport-related physical activity was also positively associated with them (*γ* = 0.11, *p* < 0.01 and *γ* = 0.17, *p* < 0.01). However, occupational physical activity was not significantly associated with them. For the worksite-level variables, scores for each workplace environment were inconsistently associated with each other. Of those variables, work rules were significantly and negatively associated with transport-related physical activity (*γ* = −0.56, *p* < 0.05), and physical activity/fitness facilities and written policies were significantly and positively associated with leisure-time physical activity (*γ* = 0.73, *p* < 0.05 and *γ* = 0.61, *p* < 0.05).

**Table 2:** Multilevel correlations among worksite- and employee-level variables (Nj = 23, Ni = 547)

Variables	*Mean (SD)*	*ICC* [95% CI]	1	2	3	4	5	6	7	8	9	10	11	12
*Worksite-level*														
1. Parking/Bike^a^	1.28 (1.0)	–	1.00	−0.16	0.23	−0.22	−0.39	0.36*	−0.09	−0.47	0.31	−0.57	−0.14	−0.02
2. Signs/Bulletin boards/ Advertisements^a^	1.15 (1.2)	–		1.00	−0.20	0.01	−0.15	−0.22*	0.49**	0.18	−0.36	−0.19	−0.07	0.02
3. Stairs/Elevator^a^	0.74 (0.6)	–			1.00	−0.35*	0.30	0.28*	−0.23	−0.12	−0.59	0.10	0.16	0.53
4. Physical activity/ Fitness facilities^a^	0.74 (1.7)	–				1.00	−0.40**	0.28	0.40	−0.04	0.33	0.22	−0.31	0.73*
5. Work rules^a^	5.04 (0.8)	–					1.00	−0.28	−0.24	−0.07	−0.43	−0.22	−0.56*	−0.20
6. Written policies^a^	0.26 (1.3)	–						1.00	0.18	0.51	0.32	−0.19	−0.12	0.61**
7. Health promotion analys^a^	1.43 (1.9)	–							1.00	0.22	0.33	−0.02	−0.07	0.02
*Employee-level*														
8. Self-regulation for physical activity^a^	24.66 (10.1)	0.10* [.01, .19]								1.00	0.73	−0.36	0.54**	0.95**
9. Self-efficacy for physical activity^a^	11.45 (3.6)	0.00 [−0.10, 0.10]								0.41**	1.00	0.27	0.73	0.97**
10. Occupational physical activity^b^	3.38 (17.8)	0.10 [−0.03, 0.23]								0.08	0.05	1.00	−0.25	−0.23
11. Transport-related physical activity^b^	9.31 (11.9)	0.15* [0.03, 0.26]								0.11**	0.17**	0.14	1.00	0.52*
12. Leisure-time physical activity^b^	7.07 (13.0)	0.04 [−0.03, 0.10]								0.40**	0.34**	0.18	0.09*	1.00

Upper triangular matrix indicates worksite-level correlations, and lower triangular matrix indicates employee-level matrix. *Nj* the number of worksites, *Ni* the number of workers, *ICC* intra-class correlation coefficient, *CI* confidence interval. ^a^At baseline. ^b^At follow-up. *p < 0.05. **p < 0.01.

Tables 3, 4, and 5 show the main results of HLM on domain-specific physical activity at follow-up, indicating different associations with each other. Because the worksite-level adjusted model (Model 5) in all three domain-specific physical activities indicated the best model fit indices (AIC = 18,629.98, 18,150.18, and 17,980.61, respectively) among the five models, we adopted Model 5 as our conclusive results. With occupational physical activity (Table 3), self-regulation for physical activity was not significantly associated (*γ*_*10*_ = 0.11 [SE = 0.16]). For workplace environments, stairs/elevator (*γ*_*03*_ = 3.80 [SE = 1.80], *p* < 0.05), physical activity/fitness (*γ*_*04*_ = 4.98 [SE = 1.09], *p* < 0.01), and written policies (*γ*_*06*_ = 2.10 [SE = 1.02], *p* < 0.05) were significantly and positively associated in the worksite-level adjusted models. A negative interaction effect between parking/bike and self-regulation for physical activity was also significant (*γ*_*11*_ = −0.43 [SE = 0.20], *p* < 0.05). With transport-related physical activity (Table 4), self-regulation for physical activity was not significantly associated (*γ*_*10*_ = −0.00 [SE = 0.06]). For workplace environments, health promotion programs were significantly and negatively associated (*γ*_*07*_ = −0.60 [SE = 0.25], *p* < 0.05). Significant and positive interaction effects of workplace environments and self-regulation were observed on physical activity/fitness facilities (*γ*_*14*_ = 0.06 [SE = 0.03], *p* < 0.05), work rules (*γ*_*15*_ = 0.28 [SE = 0.12], *p* < 0.05), and written policies (*γ*_*16*_ = 0.04 [SE = 0.01], *p* < 0.05). With leisure-time physical activity (Table 5), self-regulation for physical activity was significantly and positively associated (*γ*_*10*_ = 0.09 [SE = 0.04], *p* < 0.05). Workplace environments did not have any significant main effect. Positive interaction effects of workplace environments and self-regulation were observed on physical activity/fitness facilities (*γ*_*14*_ = 0.06 [SE = 0.03], *p* < 0.05) and written policies (*γ*_*16*_ = 0.06 [SE = 0.02], *p* < 0.05).

**Table 3:** The associations among workplace environment, self-regulation for physical activity, and occupational physical activity among white-collar workers (Nj = 23, Ni = 547)

Dependent: occupational physical activity at follow-up	Model 1 Unconditional model	Model 2 Crude random slope model	Model 3 Crude interaction model	Model 4 Employee-level adjusted model^a^	Model 5 Worksite-level adjusted model^b^
	*Coefficient* (SE)	*Coefficient* (SE)	*Coefficient* (SE)	*Coefficient* (SE)	*Coefficient* (SE)
*Employee-level*					
Self-regulation for physical activity (*γ*_*10*_)	–	0.23 (0.18)	0.13 (0.14)	0.11 (0.16)	0.11 (0.16)
*Worksite-level*					
Workplace environment					
Parking/Bike (*γ*_*01*_)	–	−0.84 (1.33)	−0.83 (1.33)	−0.81 (1.20)	−1.02 (1.41)
Signs/Bulletin boards/Advertisements (*γ*_*02*_)	–	−0.50 (1.32)	−0.49 (1.31)	−0.17 (1.21)	0.70 (0.51)
Stairs/Elevator (*γ*_*03*_)	–	0.16 (2.49)	0.12 (2.50)	0.28 (2.05)	3.80 (1.80)*
Physical activity/Fitness facilities (*γ*_*04*_)	–	1.68 (1.48)	1.68 (1.48)	1.46 (1.22)	4.98 (1.09)**
Work rules (*γ*_*05*_)	–	0.34 (2.32)	0.38 (2.39)	0.12 (2.24)	−1.25 (1.50)
Written policies (*γ*_*06*_)	–	−0.92 (0.87)	−0.89 (0.85)	−0.81 (0.83)	2.10 (1.02)*
Health promotion programs (*γ*_*07*_)	–	−0.52 (0.31)	−0.53 (0.31)	−0.53 (0.29)	−0.01 (0.16)
*Interaction effects*					
Parking × self-regulation (*γ*_*11*_)	–	–	−0.41 (0.21)*	−0.42 (0.20)*	−0.43 (0.20)*
Signs × self-regulation (*γ*_*12*_)	–	–	0.19 (0.18)	0.24 (0.16)	0.25 (0.16)
Stairs × self-regulation (*γ*_*13*_)	–	–	0.42 (0.37)	0.34 (0.32)	0.35 (0.33)
Fitness × self-regulation (*γ*_*14*_)	–	–	0.07 (0.10)	0.05 (0.09)	0.05 (0.09)
Rules × self-regulation (*γ*_*15*_)	–	–	0.04 (0.32)	0.01 (0.33)	0.00 (0.32)
Policies × self-regulation (*γ*_*16*_)	–	–	0.08 (0.10)	0.12 (0.10)	0.13 (0.10)
Programs × self-regulation (*γ*_*17*_)	–	–	−0.10 (0.07)	−0.11 (0.08)	−0.12 (0.08)
Intercept (*γ*_*00*_)	3.90 (1.54)*	3.26 (1.08)**	3.27 (1.08)**	2.87 (0.98)**	7.07 (1.71)**
Random intercept (*τ*_*00*_)	31.88 (26.72)	21.48 (12.45)	21.60 (12.37)	16.55 (13.65)	0.10 (1.39)
Random slope: self-regulation (*τ*_*11*_)	–	0.40 (0.34)	0.21 (0.11)	0.24 (0.09)**	0.25 (0.09)**
Residual variance (*e*_*ij*_)	289.41 (145.74)*	261.85 (133.85)	261.64 (133.62)	180.65 (99.80)	178.26 (96.23)
*AIC*	22,792.08	18,780.05	18,786.13	18,646.91	18,629.98

*Nj* the number of worksites, *Ni* the number of employees. *CI* confidence interval, *AIC* Akaike information criteria. Full Information Maximum Likelihood estimation (FIML) was used using Mplus 7.4. ^a^Adjusted by self-efficacy for physical activity, gender, age, employment status, occupational status, working hours, job strain, and occupational physical activity at baseline. ^b^Adjusted by employee-level covariates, worksite size, and worksite location. *p < 0.05. **p < 0.01.

**Table 4:** The associations among workplace environment, self-regulation for physical activity, and transport-related physical activity among white-collar workers (Nj = 23, Ni = 547)

Dependent: transport-related physical activity at follow-up	Model 1 Unconditional model	Model 2 Crude random slope model	Model 3 Crude interaction model	Model 4 Employee-level adjusted model^a^	Model 5 Worksite-level adjusted model^b^
	*Coefficient* (SE)	*Coefficient* (SE)	*Coefficient* (SE)	*Coefficient* (SE)	*Coefficient* (SE)
*Employee-level*					
Self-regulation for physical activity (*γ*_*10*_)	–	0.13 (0.07)	0.10 (0.02)**	−0.00 (0.06)	−0.00 (0.06)
*Worksite-level*					
Workplace environment					
Parking/Bike (*γ*_*01*_)	–	−2.98 (1.14)**	−3.00 (1.14)**	−2.48 (1.18)*	−0.61 (0.98)
Signs/Bulletin boards/Advertisements (*γ*_*02*_)	–	−1.00 (0.98)	−0.97 (0.98)	−0.82 (1.01)	0.59 (0.80)
Stairs/Elevator (*γ*_*03*_)	–	3.95 (1.96)*	3.93 (1.95)*	3.32 (1.96)	2.80 (2.01)
Physical activity/Fitness facilities (*γ*_*04*_)	–	−1.10 (0.64)	−1.11 (0.64)	−0.95 (0.70)	−0.39 (0.88)
Work rules (*γ*_*05*_)	–	−7.21 (1.97)**	−7.21 (1.97)**	−6.87 (1.94)**	−1.50 (2.89)
Written policies (*γ*_*06*_)	–	−1.44 (0.63)*	−1.47 (0.63)*	−1.34 (0.65)*	−0.72 (1.13)
Health promotion programs (*γ*_*07*_)	–	−0.03 (0.35)	−0.02 (0.35)	−0.01 (0.30)	−0.60 (0.25)*
*Interaction effects*					
Parking × self-regulation (*γ*_*11*_)	–	–	−0.01 (0.04)	0.07 (0.06)	0.07 (0.06)
Signs × self-regulation (*γ*_*12*_)	–	–	0.07 (0.04)	0.08 (0.05)	0.08 (0.05)
Stairs × self-regulation (*γ*_*13*_)	–	–	−0.00 (0.10)	0.01 (0.10)	−0.00 (0.11)
Fitness × self-regulation (*γ*_*14*_)	–	–	0.03 (0.02)	0.05 (0.03)*	0.06 (0.03)*
Rules × self-regulation (*γ*_*15*_)	–	–	0.15 (0.07)*	0.26 (0.12)*	0.28 (0.12)*
Policies × self-regulation (*γ*_*16*_)	–	–	0.06 (0.01)**	0.04 (0.01)*	0.04 (0.01)*
Programs × self-regulation (*γ*_*17*_)	–	–	−0.02 (0.01)	−0.02 (0.01)	−0.02 (0.01)
Intercept (*γ*_*00*_)	8.48 (1.21)**	10.12 (0.97)**	10.12 (0.98)**	9.37 (1.43)**	11.32 (2.05)**
Random intercept (*τ*_*00*_)	23.19 (6.85)**	11.19 (6.22)	11.20 (6.23)	12.53 (5.98)*	0.22 (1.28)
Random slope: self-regulation (*τ*_*11*_)	–	0.00 (0.02)	0.00 (0.00)	0.00 (0.01)	0.00 (0.01)
Residual variance (*e*_*ij*_)	103.26 (14.10)**	101.92 (13.89)**	101.29 (13.65)**	80.41 (12.20)**	81.84 (10.47)**
*AIC*	22,230.67	18,227.64	18,238.37	18.160.32	18,150.18

*Nj* the number of worksites, *Ni* the number of employees. *CI* confidence interval, *AIC* Akaike information criteria. Full Information Maximum Likelihood estimation (FIML) was used using Mplus 7.4. ^a^Adjusted by self-efficacy for physical activity, gender, age, employment status, occupational status, working hours, job strain, and transport-related physical activity at baseline. ^b^Adjusted by employee-level covariates, worksite size, and worksite location. *p < 0.05. **p < 0.01.

**Table 5:** The associations among workplace environment, self-regulation for physical activity, and leisure-time physical activity among white-collar workers (Nj = 23, Ni = 547)

Dependent: leisure-time physical activity at follow-up	Model 1 Unconditional model	Model 2 Crude random slope model	Model 3 Crude interaction model	Model 4 Employee-level adjusted model^a^	Model 5 Worksite-level adjusted model^b^
	*Coefficient* (SE)	*Coefficient* (SE)	*Coefficient* (SE)	*Coefficient* (SE)	*Coefficient* (SE)
*Employee -level*					
Self-regulation for physical activity (*γ*_*10*_)	–	0.57 (0.09)**	0.40 (0.04)**	0.10 (0.04)*	0.09 (0.04)*
*Worksite-level*					
Workplace environment					
Parking/Bike (*γ*_*01*_)	–	−1.53 (0.51)**	−1.55 (0.52)**	−1.29 (0.53)*	−1.05 (0.69)
Signs/Bulletin boards/Advertisements (*γ*_*02*_)	–	−0.01 (0.39)	−0.00 (0.39)	−0.11 (0.34)	0.55 (0.43)
Stairs/Elevator (*γ*_*03*_)	–	1.31 (1.40)	1.32 (1.41)	1.13 (1.47)	−0.18 (1.21)
Physical activity/Fitness facilities (*γ*_*04*_)	–	0.76 (0.31)*	0.74 (0.31)*	0.79 (0.49)	−0.03 (0.66)
Work rules (*γ*_*05*_)		−2.10 (1.11)	−2.13 (1.12)	−2.39 (1.39)	0.55 (1.28)
Written policies (*γ*_*06*_)	–	1.29 (0.25)**	1.20 (0.24)**	0.96 (0.37)*	0.99 (0.59)
Health promotion programs (*γ*_*07*_)	–	0.16 (0.11)	0.15 (0.11)	0.13 (0.16)	−0.01 (0.13)
*Interaction effects*					
Parking × self-regulation (*γ*_*11*_)	–	–	−0.24 (0.08)**	−0.08 (0.07)	−0.07 (0.08)
Signs × self-regulation (*γ*_*12*_)	–	–	0.02 (0.04)	0.04 (0.03)	0.04 (0.03)
Stairs × self-regulation (*γ*_*13*_)	–	–	0.23 (0.13)	0.08 (0.13)	0.06 (0.13)
Fitness × self-regulation (*γ*_*14*_)	–	–	0.05 (0.03)*	0.05 (0.03)	0.06 (0.03)*
Rules × self-regulation (*γ*_*15*_)			−0.21 (0.13)	0.03 (0.12)	0.04 (0.12)
Policies × self-regulation (*γ*_*16*_)	–	–	0.10 (0.02)**	0.06 (0.02)**	0.06 (0.02)**
Programs × self-regulation (*γ*_*17*_)	–	–	−0.03 (0.02)*	−0.02 (0.01)	−0.02 (0.01)
Intercept (*γ*_*00*_)	7.05 (0.85)**	7.09 (0.42)**	7.04 (0.44)**	8.53 (1.26)**	8.73 (1.44)**
Random intercept (*τ*_*00*_)	6.95 (6.91)	0.16 (0.44)	0.12 (0.71)	1.35 (.2.61)	0.03 (0.20)
Random slope: self-regulation (*τ*_*11*_)	–	0.06 (0.03)	0.00 (0.02)	0.00 (0.02)	0.00 (0.01)
Residual variance (*e*_*ij*_)	162.54 (25.71)**	129.94 (21.98)**	127.78 (21.34)**	75.04 (14.59)**	73.79 (13.93)**
*AIC*	22,291.35	18,202.44	18,198.14	17,986.55	17,980.61

*Nj* = the number of worksites, *Ni* the number of employees. *CI* confidence interval, *AIC* Akaike information criteria. Full Information Maximum Likelihood estimation (FIML) was used using Mplus 7.4. ^a^Adjusted by self-efficacy for physical activity, gender, age, employment status, occupational status, working hours, job strain, and leisure-time physical activity at baseline. ^b^Adjusted by employee-level covariates, worksite size, and worksite location. *p < 0.05. **p < 0.01.

## Discussion

This was the first longitudinal study to report multilevel effects of workplace environment and self-regulation for domain-specific physical activities among white-collar workers. The results of each analysis suggested that their effects may differ among domain-specific activities. Some of the components of workplace environments could directly increase occupational physical activity. However, transport-related and leisure-time physical activity might not be increased by environmental modification at the workplace alone without combining with enhancing self-regulation for physical activity. This may mean the necessity of conducting the studies and intervention by domains. Another implication of this study is that, to promote overall physical activity among white-collar workers, designing multi-component interventions that include both of environmental and psychological approaches might be important.

The hypothesis of the association between workplace environment and physical activity (*H1*) was supported mainly for occupational physical activity, suggesting the direct effect of workplace environment may influence physical activity at work. The effectiveness of stairs/elevator, physical activity/fitness facilities, and written policies at the workplace has been repeatedly indicated by previous studies [16, 17, 45–48]. Thus, this study may add further evidence of the association among these workplace environments and occupational physical activity, even after adjusting for worksite-level covariates (i.e., worksite size and worksite location). Possible functions of these components might be enhancing opportunities and accessibility for doing physical activity [16, 45, 46, 48, 49]. Especially, because using stairs and physical activity/fitness facilities can be directly connected to increasing the amount of physical activity at work, their associations with occupational physical activity may be stronger than “soft”’ workplace environment changes, such as mounting signs or establishing work rules. Another function might be establishing social norms at the workplace [50]. Policies and investment by employers for prompts and facilities might amplify the importance of physical activity for employees, being social, and ecological supports for doing physical activity.

The second hypotheses for self-regulation was supported for leisure-time physical activity but not for occupational/transport-related physical activities. A possible reason for the result could be difficulty to utilize their regulation for activities in occupational/transport-related settings. In most work time, employees must pay attention to their own jobs, and most activities related to jobs basically occur incidentally [51]. Therefore, most employees may have difficulty using their psychological functions for occupational physical activity within working time. Transport-related physical activity is also largely influenced by environmental factors at both workplaces and communities, and most employees may establish their commuting habits. Therefore, there may be few choices to commute and little need to actively regulate their strategies for transport-related activities. On the other hand, workers could thoroughly utilize their cognitive and behavioral regulation for physical activity in leisure time. Considering that there was a significant positive relationship between self-efficacy for physical activity and physical activity in the workday [23, 24], functions of self-efficacy and self-regulation might also differ for each domain of physical activities.

It is interesting that the effects of workplace environments may spill over into outside of the worksite through boosting the associations between self-regulation and physical activity (*H3*). For transport-related physical activity, strategies of self-regulation (*γ*_*10*_ = −0.00) are ineffective themselves (*H2*) but effective only when physical activity/fitness (*γ*_*14*_ = 0.06, *p* < 0.05), work rules (*γ*_*15*_ = 0.28, *p* < 0.05), and written policies (*γ*_*16*_ = 0.04, *p* < 0.05) are well facilitated at the workplace. For leisure-time physical activity, strategies of regulation are more effective when physical activity/fitness (*γ*_*14*_ = 0.06, *p* < 0.05) and written policies (*γ*_*16*_ = 0.06, *p* < 0.05) are well facilitated. These results have a practical implication for future multi-component interventions that either environmental modification or psychological approach alone is insufficient to promote transport-related and leisure-time physical activity among white-collar workers. These workplace environments include not only internal physical environments at the workplace but also external and social environments. Therefore, they might be useful for employees to be more active when employees plan physical activity outside of the workplace. For instance, setting a goal to increase transport-related physical activity during lunch break can be more effective if employers set rules that enable employees to go out or if employers have a written policy that encourages employees to walk around the workplace. Another possible mechanism is that they enhance awareness and build social norms for physical activity [52]. Internalized awareness and norms could spill over into one’s transport-related and leisure-time activities and entire lifestyle among white-collar workers. Although the question of how these components moderate the association between self-regulation for physical activity and physical activity was not the focus of the present study, it should be investigated in future studies. In addition, we could only investigate one possible model that workplace environment would exaggerate the effect of self-regulation for physical activity on physical activity. The other possible relationships among two-level factors, such as mediation model and subgroup effects should be investigated in future studies.

Incidentally, some associations were negative and significant: the direct association between health promotion programs and transport-related physical activity and the interaction effect of parking/bike and self-regulation for physical activity on occupational physical activity. These inverse associations were not consistent with our expectation. Although the reasons for negative impact of workplace environments for physical activity were unknown in the present study, some components of workplace environment could be adverse for specific domains. Not only possible benefits but also potential adverse effects of workplace environment should be investigated in the future.

There are several limitations to this longitudinal study. First, the response rate of worksite representatives that agreed to participate was not high (46.9%), which can cause selection bias resulting in an underestimation of the associations. In addition, participants who were dropped were significantly younger. Although we addressed attrition at follow-up by imputation of FIML, it may cause underestimation. Second, all employee-level variables and some worksite-level variables were measured by self-report questionnaires. Measured values contained information bias and measurement errors. It has been repeatedly indicated that self-reported physical activity was often distorted with actual physical activity and often overestimated [53]. In addition, light physical activity could not be measured due to the questionnaire. Furthermore, distribution of the three domains of activities was skewed and might not much suitable for HLM. Third, the results could be distorted by confounding factors that could not be considered in this study, such as other types of job stressors [54] and environmental determinants outside the workplace. Finally, the samples were not extracted at random and were from a restricted area in Japan. Thus, there are limitations to the generalizability of the results. These limitations need to be addressed by conducting higher-quality studies and randomized controlled trials.

## Conclusion

In summary, the components of workplace environments such as physical activity/fitness facilities, written policies, signs for stair use at stairs and elevators, and work rules and self-regulation for physical activity may be effective to promote three domain-specific physical activities directly or by augmenting the positive associations between self-regulation for physical activity and physical activity. Those effects may differ by activity domain; occupational activities may be increased by workplace environments while transport-related and leisure-time activities may also be increased by the interactions of workplace environments and self-regulation. This study has practical implications for designing multi-component interventions that include both of environmental and psychological approaches to increase effect sizes to promote overall physical activity.

## Abbreviations

AIC: Akaike information criteria
EAT: Environmental assessment tool
FIML: Full information maximum likelihood
GPAQ: Global physical activity questionnaire
HLM: Hierarchical linear modeling
JCQ: Job content questionnaire
PASR: Physical activity self-regulation
STROBE: Strengthening the reporting of observational studies in epidemiology
